# Is Low Serum Vitamin D Associated with Early Dental Implant Failure? A Retrospective Evaluation on 1625 Implants Placed in 822 Patients

**DOI:** 10.1155/2016/5319718

**Published:** 2016-09-22

**Authors:** Francesco Mangano, Carmen Mortellaro, Natale Mangano, Carlo Mangano

**Affiliations:** ^1^Department of Surgical and Morphological Science, Dental School, University of Insubria, 21100 Varese, Italy; ^2^Department of Health Sciences, University of Eastern Piedmont, 28100 Novara, Italy; ^3^Division of Endocrinology and Metabolism, Moriggia Pelascini Hospital, 22015 Gravedona ed Uniti, Italy; ^4^Department of Dental Sciences, University Vita Salute San Raffaele, 20132 Milan, Italy

## Abstract

*Aim*. To investigate whether there is a correlation between early dental implant failure and low serum levels of vitamin D.* Methods*. All patients treated with dental implants in a single centre, in the period 2003–2015, were considered for enrollment in this study. The main outcome was early implant failure. The influence of patient-related variables on implant survival was calculated using the Chi-square test.* Results*. 822 patients treated with 1625 implants were selected for this study; 27 early failures (3.2%) were recorded. There was no link between gender, age, smoking, history of periodontitis, and an increased incidence of early failures. Statistical analysis reported 9 early failures (2.2%) in patients with serum levels of vitamin D > 30 ng/mL, 16 early failures (3.9%) in patients with levels between 10 and 30 ng/mL, and 2 early failures (9.0%) in patients with levels <10 ng/mL. Although there was an increasing trend in the incidence of early implant failures with the worsening of vitamin D deficiency, the difference between these 3 groups was not statistically significant (*P* = 0.15).* Conclusions*. This study failed in proving an effective link between low serum levels of vitamin D and an increased risk of early implant failure. Further studies are needed to investigate this topic.

## 1. Introduction

Dental implants are now a reliable solution for the functional and esthetic rehabilitation of partially and completely edentulous patients; this has been demonstrated by long-term clinical trials, with survival rates of greater than 95% [1–3].

In order to achieve long-term survival, osseointegration of the dental implant needs to occur; that is, a direct connection must be established between the bone and the implant surface, without the interposition of fibrous tissue [4]; once established, this close bond must be maintained over time, resulting in a clinically asymptomatic fixation of the implant under functional load [5]. Osseointegration is a complex phenomenon and depends on many factors; some are related to the implant (material, macroscopic design, and implant surface), others to the surgical-prosthetic protocol (surgical technique, loading conditions, and time), and others to the patient (quantity/quality of bone at the receiving site and the host response) [4, 5].

Although survival rates of dental implants are now high, there still remains a seemingly unavoidable number of failures: either cases in which correctly placed implants do not integrate with the bone or cases of peri-implant tissue infection [6, 7]. To be specific, failure to osseointegrate and peri-implantitis are the most frequent causes of early implant failure [3, 6, 7]. Such events occur during the early stages of healing (within 2-3 months of implantation) and therefore before the implant is functionally loaded with the prosthetic restoration; these failures are unevenly distributed within the general population and tend to occur in some subjects in particular. In these individuals multiple or repeated failures over time are possible [6, 7]. Early failures occur even when optimal materials are used, surgical protocols are strictly followed, and the quantity/quality of bone at the recipient site is sufficient [6–8]. All these observations would suggest the existence of specific patient-related risk factors; this prompts an investigation into the regulatory mechanisms controlling bone metabolism, bone remodelling, and bone turnover [9, 10].

Vitamin D plays a fundamental role in bone metabolism [11–13]. It is a fat-soluble vitamin which promotes the absorption of calcium in the intestine and regulates calcium and phosphate homoeostasis in the tissues and it is a fundamental element in the mineralization of bones and teeth [11–13]. It also acts as a hormone and is vital for the health of the blood vessels and the brain [14, 15]. It has been demonstrated that vitamin D plays a crucial role in the health of the cardiovascular tract [16], the immune system [17], and the respiratory tract [18, 19].

Vitamin D in an inactive form (cholecalciferol or vitamin D3) is ingested or produced in the skin on exposure to sunlight [11, 12]. This inactive form undergoes double hydroxylation in the liver and the kidneys and is thereby transformed into its active form, known as either calcitriol or 1,25-dihydroxyvitamin D3 [11, 12]. This active form exerts its action on various tissues by binding to the vitamin D receptors and regulating the transcription of specific target genes [12–23].

Serum levels of vitamin D in the 25(OH)D form are the most accurate way of determining vitamin D status: a subject with <10 ng/mL is considered to be vitamin D deficient; one with 10–30 ng/mL is considered to have low levels of vitamin D. The optimal blood level of vitamin D is a value >30 ng/mL [12, 13]. Vitamin D deficiency is high in the general population [12]: in Italy, for example, it is estimated that about 80% of people can be deficient, particularly in the northern regions where exposure to the sun is lower [20]. This deficiency increases with age and encompasses the majority of the elderly population of Italy who are not taking vitamin D supplements [20]. Until a few years ago, the guidelines estimated that the daily intake of vitamin D required to maintain adequate blood levels was 200 IU (5 mcg) in adults aged between 19 and 50, 400 IU (10 mcg) in adults aged between 51 and 69, and at least 600 IU (15 mcg) in those over 70 [12, 13]. These guidelines have now been revised upwards and it is currently believed that the amount of vitamin D which should be taken daily is 2000 IU (50 mcg) and up to 4000 (100 mcg) in the case of, for example, pregnant women [12, 13].

There is now substantial literature on the negative effects of low levels of vitamin D, especially in severely compromised patients: vitamin D deficiency seems to be associated with increased mortality, cardiovascular events, and reduced functioning of the immune and musculoskeletal systems [15–19, 21–23]. On the other hand, normalizing levels of vitamin D can lead to substantial benefits for critically ill patients, with effects on the muscles, the respiratory system, the heart, and the immune system [18, 21, 23].

Despite the importance of vitamin D and its effects on bone metabolism [11, 12] few studies have, to date, investigated the effects of its depletion on the osseointegration of dental implants [9, 24–35]: almost all these studies have been done on animal models [24–32] and very few on humans [33–35]. The purpose of this retrospective study was therefore to investigate any possible correlation between low blood levels of vitamin D and early implant failure (failure occurring in the four months prior to the full restoration of the implant, because of a lack of osseointegration or because of infection).

## 2. Materials and Methods

### 2.1. Data Collection

All patients who had been treated with Morse-taper connection dental implants (Leone® Implant System, Florence, Italy) [3, 8] inserted to support fixed prosthetic restorations in one single dental centre (Gravedona, Como, Italy), in the period between January 2003 and December 2015, were evaluated for possible enrollment into this retrospective study. Patients were enrolled into the study if they were over 18 years of age, had good oral and general health, and had not undergone bone regenerative therapy prior to implant placement. The exclusion criteria were incomplete medical records, the presence of specific systemic diseases (uncontrolled diabetes mellitus, immunodeficient states, and bleeding disorders), and the abuse of alcohol and drugs; patients undergoing radiotherapy and chemotherapy and those who were pregnant were also excluded. All the data used for the study were obtained from the medical records of the patients enrolled. The patient data was evaluated; this included gender (male or female), age at time of surgery, history of chronic periodontal disease, smoking habits, and serum vitamin D levels. Vitamin D levels were taken from blood tests, which had been requested two weeks prior to surgery. The medical records also contained a range of information as regards the implant or implants, that is, their site (maxilla or mandible), location (incisor, canine, premolar, and molar), the length and diameter of the implant, the type of prosthetic restoration, and the loading conditions. Lastly, the medical records contained all the information on any implant failure. These included their cause (lack of osseointegration in the absence of infection, infection of the peri-implant tissues or peri-implantitis, or implant failure due to progressive bone loss caused by to prosthetic overload). It also included their classification: early failure, occurring in the early healing period, that is, the four months after implant placement, prior to the placement of restoration and loading, or late failure, occurring after loading. There were also details of any possible biological complications (peri-implant mucositis and peri-implantitis) and/or prosthetic complications (mechanical and/or technical).

### 2.2. Insertion Protocol for the Implants

All implants were inserted under the same strict protocol by the same specialist (C. M.) who had 25 years' experience in implant dentistry, in the period between January 2003 and December 2015. The implants were inserted after raising a full thickness mucoperiosteal flap; the implant site preparation and implant placement were performed in compliance with modern surgical protocols and in accordance with the manufacturer's instructions. After placement, cover screw was positioned and the implants were submerged. Immediately after positioning, patients were prescribed antibiotic coverage with 2 g of amoxicillin (or 600 mg of clindamycin in patients allergic to penicillins) for 6 days. Postoperative pain was controlled with nimesulide 100 mg daily for 2 days. Patients were given detailed instructions on oral hygiene and were prescribed chlorhexidine 0.12% mouth rinse twice a day for 6 days. The patients were recalled for suture removal after 10 days. The implants were left to heal submerged for a total period of 4 months, to allow undisturbed healing and achieve osseointegration. After 4 months of undisturbed healing, the patient was recalled for the implant to be uncovered. The cover screw was replaced with the healing abutment, and sutures were positioned. Two weeks later, the sutures were removed and an impression was taken for the manufacture of the temporary restoration. The temporary restoration was maintained* in situ* for 3 months, in order to monitor the response of the implant, as well as the peri-implant tissues, to masticatory load; at the end of this period, the temporary restoration was replaced with the final restoration. The final restorations were metal porcelain, cemented with a zinc oxide-eugenol cement. A periapical radiograph was taken to check on the sealing of the restoration. All patients were included in a follow-up protocol with an annual check-up at one of the scheduled professional oral hygiene sessions.

### 2.3. Primary Outcome

Early implant failure, occurring within 4 months after implant placement and therefore prior to placement of the prosthetic restoration and the functional load of the implant, was the primary outcome studied. Early implant failures were divided into two different categories: (a) early failures due to lack of osseointegration and subsequent implant mobility, in the absence of clinical signs of infection; (b) early failures due to infection of the bone tissue around the implant, with inflammation (peri-implantitis) of peri-implant tissues and the presence of fistula, pain, swelling, pus and/or exudate, pocket depth >6 mm with bleeding, and marginal bone resorption >2.5 mm.

### 2.4. Statistical Analysis

All the data retrieved from the individual medical records were recorded on a generic spreadsheet (Excel®, Microsoft Office, Redmond, MA, USA) which was used for the descriptive, qualitative, and quantitative analyses. The mean, standard deviation, median, and confidence intervals were calculated for the quantitative variables (e.g., patients' age and vitamin D levels in serum). A patient-based technique was used to calculate implant survival. In this analysis, the “event” was implant failure: thus in patients receiving more than one implant, the occurrence of even a single implant failure led to the patient being classified as a “failure.” The influence of different variables on implant survival was taken into consideration: gender (male or female), age at time of surgery (three age groups were examined: <40, 40–60 years, and >60 years), smoking habits (regardless of the actual number of cigarettes smoked), a history of chronic periodontitis [36], and serum levels of vitamin D. In the analysis of serum levels of vitamin D, three classes of patients were considered: severely deficient patients (serum vitamin D <10 ng/mL), patients with low levels (serum vitamin D between 10 and 30 ng/mL), and patients with adequate levels (serum vitamin D >30 ng/mL). The influence of each of these variables on implant survival was calculated using the Chi square test. The significance level was set at 0.05. The overall implant survival, the survival within the different groups, and the analysis of the influence of the different variables on survival were all made using dedicated statistical analysis software (SPSS 17.0®, SPSS Inc., Chicago, IL, USA).

## 3. Results

Of the 915 patients originally evaluated for enrollment in this study, 93 presented with conditions corresponding to the exclusion criteria and were therefore excluded from the assessment. By contrast, 822 patients (mean age 57.3 ± 14.2 years; median age 58; range 18–90; and 95% CI, 56.3–58.2), receiving 1625 implants, did not have any of the conditions contained in the exclusion criteria and were therefore enrolled into this retrospective study. The distribution of patients by groups, with relative incidence of failures, was reported in Table 1. In total, 27 early failures were recorded (19 due to failure of osseointegration and 8 due to peri-implant tissue infection), with an overall incidence of 3.2%. No differences were observed in the incidence of early failures between males and females (*P* = 0.97) nor according to age at time of surgery (*P* = 0.98). Although the percentage of early failures in smokers was slightly higher than that detected in nonsmokers, there was no statistically significant difference (*P* = 0.56) between these two groups of patients. The same was true for patients with a history of periodontal disease; they displayed a slightly higher incidence of early failures than patients who had not been affected by periodontitis, but this difference was not significant (*P* = 0.73). The average serum level of vitamin D in the general population was 29.9 ng/mL (±12.1; median 29; range 5–73; and 95% CI, 29.1–30.7). In patients in whom early implant failure occurred, the average serum level of vitamin D was 25.5 (±13.2; median 24; range 8–55; and 95% CI, 20.6–30.4). Statistical analysis reported a rather low incidence of early failures (2.2%) in patients with blood vitamin D levels >30 ng/mL. The incidence of early failure was almost double in patients with insufficient serum levels of vitamin D (10–30 ng/mL) and became even higher (9.0%) in patients with serious vitamin D deficiency. Although the statistical analysis revealed a trend toward an increased incidence of failure in patients with severe vitamin D deficiency, the analysis did not reveal a statistically significant difference (*P* = 0.15) in the incidence of early implant failure in these three groups of patients. Similar results (*P* = 0.14) were obtained comparing the incidence of failures in the group of severely deficient patients (2/22: 9.0%) with the incidence of failures in all other patients (25/800: 3.1%). Finally, the statistical analysis did not reveal a significant difference (*P* = 0.13) when comparing the incidence of failures in the group of patients with serum vitamin D levels >30 ng/mL (9/394: 2.2%) with the incidence of failures in all other patients (18/428: 4.2%). The details of early failure were reported in Table 2.

## 4. Discussion

A relatively small number of experimental studies has attempted to investigate the effects of vitamin D on the osseointegration of dental implants [24–32]. The majority of these studies would appear to indicate a positive effect of vitamin D on osseointegration, but it is not yet entirely clear whether supplementation would promote the healing of peri-implant bone tissue clinically [24, 33–35].

A recent review of the literature on animal studies has shown that vitamin D supplementation can stimulate new bone formation and increase the contact between the bone and the surface of titanium implants [24]. Specifically, Kelly et al. demonstrated that vitamin D deficiency could significantly compromise the establishment of osseointegration of Ti6Al4V implants in rats [25]. Similar results were reported by Dvorak et al. [26]. In an experimental study on ovariectomized rats, the authors demonstrated that vitamin D deficiency could impair the formation of peri-implant bone; the normalization of blood levels via supplementation of vitamin D stimulated new bone formation [26]. Similar results were reported by Zhou et al., who found an increase in osseointegration in osteoporotic rats given vitamin D supplements [27], and Wu et al., who demonstrated an increase in the percentage of contact between bone and implant in diabetic rats given vitamin D supplements [28]. Finally, Liu et al. reported that the administration of vitamin D could increase the fixation of dental implants in mice suffering from chronic kidney disease [29].

A further possibility for study, in order to understand the effects of the administration of vitamin D on bone healing of the peri-implant tissues, is that of coating the implant surface with vitamin D [30–32]. Salomό-Coll et al. evaluated the effect of the topical application of vitamin D to the surface of implants inserted in postextraction sockets in dogs, with histological and histomorphometric analyses of tissues removed at 12 weeks [30]. Topical application of vitamin D increased the percentage of bone to implant contact of 10% [30]. Similarly promising results were reported by Cho et al. in a histological and histomorphometric study on rabbits, where the coating of anodized implant surfaces with a solution of poly(D,L-lactide-co-glycolide) PLGA and 1*α*,25-dihydroxyvitamin D3 (1*α*,25-(OH)2D3) stimulated the apposition of new bone on fixtures [31]. Finally, in a further experimental work in rabbits, implants with a surface coated in 1,25-(OH)2D3 have shown an improved tendency to osseointegrate compared to noncoated implants; however, this difference was not statistically significant [32].

Unfortunately, very few clinical studies have so far investigated the effects of vitamin D deficiency on osseointegration and on bone regeneration in dentistry [33–35]. This is probably due to the fact that there are many factors which can determine the success or failure of dental implants; the attention of clinicians has been mostly focused on drawing up surgical and prosthetic protocols and identifying new materials and implant surfaces to improve osseointegration, rather than on the analysis of patient-related risk factors [6–9]. In a recent clinical work, Alvim-Pereira et al. found no relationship between polymorphism of the vitamin D receptor and implant failure [33]. In a randomized, controlled, double-blind study, Schulze-Späte et al. investigated the effects of supplementation with a combination of vitamin D3 (5000 IU) and calcium (600 mg) on the formation of new bone following maxillary sinus lift [34]. Ten patients were assigned to the test group and given vitamin D and calcium; ten other patients were assigned to the control group and received only calcium [34]. Six to eight months after surgery for bone regeneration, bone samples were taken for histological analysis during implant placement [34]. Although supplementation with vitamin D3 would have increased the serum levels of vitamin D with potentially positive effects on bone remodelling at the cellular level, no statistically significant difference was demonstrated between the two groups at the histological level [34].

The results of our study would appear to suggest that a severe deficiency of vitamin D in the blood might be related to an increase in the incidence of early implant failure. In fact, the incidence of early implant failure was rather low (2.2%) in patients with normalized levels of vitamin D in the blood (>30 ng/mL), rose to almost double (3.9%) in patients with insufficient serum levels (10–30 ng/mL), and were rather high (9.0%) in patients characterized by severe deficiency states. However, despite the tendency to an increased incidence of early failure in patients characterized by deficiency states, the differences between the three groups of patients were not statistically significant (*P* = 0.15).

Our study also confirms that the serum values of vitamin D in the local population are rather low: we found that the proportion of patients with insufficient levels was 49.4% and that the percentage with a severe deficiency was 2.7%. The percentage of patients with adequate levels was 47.9%. This is not surprising, as most of the patients treated came from northern Italy and southern Switzerland, regions where exposure to sunlight is somewhat reduced for long periods of the year. In the light of this, the administration of vitamin D in the weeks prior to placement of a dental implant could be useful, particularly in patients with severe deficiency states; in these patients, vitamin D supplementation should be maintained for the whole life, in order to guarantee a good remodelling of the bone around the implant.

This study has the distinction of being one of the first clinical studies carried out on a large number of patients to investigate the possibility of an association between low blood levels of vitamin D and the incidence of early failure in implantology. By restricting the analysis to early failures, occurring in the first period of healing and therefore prior to placement of the prosthetic restoration, we were able to focus our research and avoid a range of factors (linked to the restoration itself and the prosthetic load) which could have confused the issue in the study. It is, in fact, well known that implant survival and thus osseointegration depend on a large number of factors (related to the surgical and prosthetic protocol, the materials used, and lastly the patient) [4, 5] and it can be difficult to identify which of them might be determining the success or failure of the treatment [7]. In order to avoid this and to limit the confounding factors, the same materials were used for all the patients in this study (the same implant system for all the patients) [1, 3, 8]. In addition, the same surgical protocol was used, involving submerged healing in the absence of prosthetic loading [3]. Thus the only possible confounding factors were the different quantity and quality of bone at the implant receiving sites and the patients' responses: these are unavoidable factors. However, some of those categories of patients most at risk of implant failure (patients undergoing bone regeneration to create the conditions for the positioning of the implant fixtures or those with particular medical conditions which might increase the risk of treatment failure) were excluded in the present study.

This study has limits. It is a retrospective work, in which the number of patients having a severe deficiency of vitamin D in the blood was low (only 22); thus the presence of even just one less failure in this group would have led to quite different results. It is possible that some residual confounding may have biased the association between vitamin D and implant failures that we observed. For instance, this study did not investigate the influence of other patient-related factors (e.g., the bone quality) which can affect implant survival in the period immediately following implant placement. In addition, if subjects with low levels of vitamin D were also likely to receive more than 1 implant, their risk of being classified as “failures” may increase. However, no patient in this study experienced more than 1 failure, and the probability of implant failure was not higher (1.5% versus 2.1%) in presence of another implant. Therefore, randomized, controlled clinical trials are needed to confirm the presence of an association between low serum levels of vitamin D and an increase in the incidence of early failure in implantology. It would be appropriate to assess whether supplementation of vitamin D in the weeks before the operation could lead to a reduction in early failures, whether due to lack of osseointegration or implant infection. Further scientific studies with an appropriate design and a more rigorous statistical analysis will therefore be required in order to thoroughly investigate this issue.

## 5. Conclusions

Until now, very few studies, and those mainly on animal models, have involved assessing the influence of blood levels of vitamin D levels on the osseointegration of dental implants. Although most of these studies have shown that the administration of vitamin D can improve the healing of the peri-implant bone tissue, it is not yet clear whether vitamin D supplements can promote the osseointegration of dental implants. Our retrospective clinical study aimed to investigate if there is a link between low levels of vitamin D in the blood and an increased risk of early implant failures. Although the incidence of early implant failures was higher in patients with low serum levels of vitamin D, our study failed in proving an effective link between low levels of vitamin D in the blood and an increased risk of early implant failure. Further higher level studies (prospective controlled trials or, even better, randomized controlled clinical trials) with a more rigorous statistical analysis are therefore needed to investigate this issue. If an association between low serum levels of vitamin D and higher risk of early implant failure could be demonstrated, the clinician could give a set dose of vitamin D in the weeks before surgery, in order to normalize serum levels and obtain a positive effect on the healing process.

## Figures and Tables

**Table 1:** Distribution of patients (by gender, age at surgery, smoking habit, history of periodontal disease, and vitamin D levels in serum), related failures, survival rate within groups, and differences between groups (Chi square test).

	Number of patients	Early failures	Incidence of early failures	*P* value^*∗*^
Gender				
Males	424	14	3.3%	0.97
Females	398	13	3.2%	
Age at surgery				
<40 years	92	3	3.2%	0.98
40–60 years	380	12	3.1%	
>60 years	350	12	3.4%	
Smoking habit				
Yes	261	10	3.8%	0.56
No	561	17	3.0%	
History of periodontal disease				
Yes	279	10	3.5%	0.73
No	543	17	3.1%	
Vitamin D level in serum				
<10 ng/mL	22	2	9.0%	0.15
10–30 ng/mL	406	16	3.9%	
>30 ng/mL	394	9	2.2%	

Statistically significant difference *P* < 0.05.

^*∗*^ *P* value = Chi square test.

**Table 2:** Details of all early implant failures.

Patient	Gender	Age at surgery	Smoking habit	History of periodontal disease	Vitamin D level in serum	Type of failure	Position of the failed implant
1	M	66	Yes	No	20 ng/mL	FO	#24
2	M	55	No	No	35 ng/mL	FO	#25
3	F	42	No	No	55 ng/mL	FO	#14
4	M	38	No	Yes	48 ng/mL	FO	#15
5	F	57	Yes	Yes	45 ng/mL	PI	#15
6	F	76	No	Yes	24 ng/mL	FO	#26
7	M	45	Yes	No	55 ng/mL	FO	#17
8	M	56	No	No	10 ng/mL	PI	#17
9	F	58	No	No	8 ng/mL	FO	#26
10	M	62	Yes	Yes	22 ng/mL	FO	#25
11	M	45	No	No	12 ng/mL	FO	#13
12	M	44	No	No	28 ng/mL	FO	#21
13	F	38	No	No	25 ng/mL	PI	#16
14	M	69	Yes	Yes	24 ng/mL	PI	#24
15	F	74	No	No	25 ng/mL	FO	#26
16	M	70	Yes	No	18 ng/mL	FO	#27
17	M	69	No	No	33 ng/mL	FO	#46
18	F	58	Yes	No	32 ng/mL	FO	#46
19	F	46	Yes	Yes	12 ng/mL	PI	#35
20	M	52	No	Yes	9 ng/mL	PI	#33
21	F	43	No	Yes	29 ng/mL	FO	#46
22	F	26	No	No	33 ng/mL	FO	#37
23	F	68	Yes	No	12 ng/mL	PI	#42
24	M	78	Yes	No	15 ng/mL	FO	#35
25	M	65	No	No	22 ng/mL	FO	#44
26	F	66	No	Yes	20 ng/mL	FO	#36
27	F	70	No	Yes	18 ng/mL	PI	#45
